# Evaluating the performance of ACR, SLICC and EULAR/ACR classification criteria in childhood onset systemic lupus erythematosus

**DOI:** 10.1186/s12969-021-00619-w

**Published:** 2021-09-09

**Authors:** Reem Abdwani, Eiman Masroori, Eiman Abdullah, Safiya Al Abrawi, Ibrahim Al-Zakwani

**Affiliations:** 1 Department of Child Health, College of Medicine & Health Sciences, Sultan Qaboos University, Muscat, Oman; 2 Department of Pediatrics, Royal Hospital, Muscat, Oman; 3 Department of Pharmacology & Clinical Pharmacy, College of Medicine & Health Sciences, Sultan Qaboos University, Muscat, Oman; 4 Gulf Health Research, Muscat, Oman

## Abstract

**Background:**

The ACR 1997, SLICC 2012 and EULAR/ACR 2019 classification criteria were validated based on adult patients. To date, there are no classification criteria specific for children with SLE. The aim of the study is to compare the performance characteristics among the three SLE classification criteria (ACR-1997, SLICC-2012 and EULAR/ACR-2019) in childhood onset SLE (cSLE) cohort of Arab ethnicity from Oman.

**Methods:**

We conducted a retrospective multicenter study in Oman of cSLE patients as cases and patients with other rheumatic disease with a positive ANA titer as controls. The cSLE cases recruited were children diagnosed with SLE before 13 years of age. Data was retrospectively collected to establish the ACR-1997, SLICC-2012 and EULAR/ACR-2019 criteria fulfilled at first visit, first year follow up and last follow up.

**Results:**

Study population included 113 cSLE cases (mean age at diagnosis of 7.3 ± 3.4 years with disease duration of 6.1 ± 4.6 years) and 51 controls (mean age at diagnosis 5.0 ± 3.4 with disease duration 5.7 ± 3.9). The cSLE cases had higher frequency of familial SLE than controls (38% vs 7.8%; *p* < 0.001). The performance measures demonstrated that EULAR/ACR-2019 criteria had the highest sensitivity (81, 88, 89%) compared to ACR 1997 (49, 57, 66%) and SLICC 2012 (76, 84,86%); while the ACR 1997 had the highest specificity (96%) compared to SLICC 2012 (94%) and EULAR/ACR 2019 (90%) at first visit, first year and last assessment. When we increased the threshold score to ≥13 rather than the traditional score ≥ 10 for ACR/EULAR 2019, there was increased specificity (96%) at the expense of lower sensitivity (76, 83, and 84%) at first visit, first year and last assessment.

**Conclusion:**

In this cSLE population, EULAR/ACR 2019 scored better at initial presentation, first year and last assessment follow up. Further multinational studies are needed to validate the appropriate cut off score for the newly proposed ACR/EULAR 2019 classification criteria in cSLE to increase early sensitivity and specificity for cSLE classification.

## Background

Systemic lupus erythematosus (SLE) is a complex multisystem autoimmune disease with a diverse clinical phenotype that affects all ages and ethnicities. Childhood onset SLE (cSLE) represents around 20% of SLE cases and is often associated with a higher disease severity, morbidity and mortality than reported for adult onset disease [1–3]. Regardless of age of disease onset, the diagnosis is often challenging due to variability in disease expression which can mimic other autoimmune, infectious or hematologic diseases. The diagnosis of SLE relies on a set of clinical and laboratory findings after excluding alternative diagnosis. Hence, various classification criteria have been developed to ensure the inclusion of homogeneous groups of patient in clinical trials and epidemiological studies. However, these criteria are often used in clinical practice to aid diagnosis [4].

The 1982 American College of Rheumatology (ACR) classification criteria for SLE and their 1997 revision have shaped our understanding of SLE and has been widely used in lupus research for decades [5]. Some limitations of the ACR classification raise concerns, including inclusion of the most typical features only, possible duplication of highly correlated cutaneous manifestations, omission of many neurological manifestations, inadequate quantification of urine protein by dipstick, omission of low complement levels, lack of standardization for autoantibody detection and classification as SLE for patients who do not satisfy the immunological disorder criterion [6–9].

The Systemic Lupus International Collaborating Clinics (SLICC) classification criteria was introduced in 2012 [10]. The main changes proposed in relation to the 1997 ACR criteria [5] were redefinition of the four cutaneous criteria, inclusion of the urine protein: creatinine ratio, expansion of neurological criteria, separation of cytopenias and autoantibodies as individual criteria and the inclusion of alopecia and hypocomplementaemia. To be classified as SLE, the patient must meet at least 4 of 17 criteria, including at least 1 clinical and 1 immunological criterion or documented lupus nephritis (LN) with antinuclear antibodies (ANA) and/or anti-dsDNA. SLICC criteria is reported to have partially succeeded in improved performance over the ACR criteria, in that it had increased sensitivity (92–99% vs 77–91%) at the expense of reduced specificity (74–88% vs 91–96%) [9–11]. Both the ACR and SLICC criteria classify SLE based a simple count of the number of criteria present without taking into account disease severity and heterogeneity.

A novel SLE classification criteria jointly supported by European League Against Rheumatism (EULAR) and ACR was introduced in 2019, is based on two concepts, namely the presence of ANA as an entry criterion coupled with a scoring system based on variably weighed clinical and laboratory features [12]. The patient is classified as SLE if the total score is equal to or greater than 10. Performance characteristics in adult onset SLE found a sensitivity of EULAR/ACR to be similar to the SLICC criteria (98% vs 95% for SLICC and 85% for ACR 1997) while maintaining the specificity of the ACR 1997 criteria (97% vs 95% for ACR 1997 and 90% for SLICC) [12].

To date, studies comparing the performance of various classification criteria in cSLE are limited [13–19]. Most of the studies reported are from North America and Europe, however to date, none of the studies reported are of children of Arab ethnicity. Knowing the influence of ethnicity on the susceptibility of SLE, the aim of the current study is to compare the performance characteristics of ACR-1997, SLICC-2012 and EULAR/ACR-2019 classification criteria to identify children with diagnosis of SLE at first visit, first year follow up and last assessment in a cohort of patients of Arab ethnicity from Oman.

## Methods

The study included patients diagnosed with cSLE from both pediatric rheumatology centers in Oman over the past 10 years (2010–2020). All patients included had disease onset before 13 years of age which is the cut off age of transitioning to adult service as in many Arab countries due to cultural variation. In total, 51 patients were enrolled from Sultan Qaboos University Hospital (SQUH), while 63 patients were enrolled from Royal Hospital, both main referral centers for the country located in Muscat. The controls consisted of patients who were followed in both pediatric rheumatology centers for at least 1 year with other rheumatic diseases with ANA positivity at ≥1:80 serum dilution. To be included as a case and control, the diagnosis had to be confirmed by a consultant pediatric rheumatologist with over 10 years of clinical1 one year follow up and were excluded if they had undifferentiated diagnosis or were followed up for < 1 year.

A set of defined variables were obtained for cases and controls from the hospital electronic information system on standardized forms. Data collection included demographic, clinical and laboratory parameters. Demographic characteristics included age at disease onset, gender, disease duration, and geographic region of origin. The data of history, physical examination and laboratory features were obtained from the first clinic visit, at 1 year follow up and last follow up visit. Criteria definition were those provided by the 1997-ACR [5], 2012-SLICC [10], and 2019- EULAR/ACR [12] criteria. Cases were classified as SLE if met ≥4 criteria for ACR 1997, ≥ 4 criteria or biopsy proven lupus nephritis with ANA or anti-dsDNA for SLICC and had a total score ≥ 10 with at least one clinical criterion for EULAR/ACR criteria.

The immunological parameters were all performed at our local institution included ANA, which were determined by immunofluorescence using Hep-2 cells as substrate with a cut of > 1:80; autoantibodies including anti–double stranded DNA (anti-dsDNA), anti-extractable nuclear antigen (anti-ENA) profile included (Ro, La, Smith, and ribonuclear proteins (RNP), as well as antiphospholipid antibodies (aCL) were measured qualitatively using enzyme linked immunosorbent assay (ELISA) technique. Autoantibodies were considered positive if the value was above the cut-offs for the laboratory at least in one determination during the follow-up period, except for anti-cardiolipin antibodies, which were considered present if there was two positive occasions 12 weeks apart. aCL cut-off value of 20 MPL or GPL for ACR1997 and SLICC criteria, and a cut-off value of 40 MPL or GPL for EULAR/ACR criteria set. Lupus anticoagulant was determined by the dilute Russell’s viper venom time with confirmatory testing. Other laboratory tests performed included direct Coombs test, levels of complement proteins C3 and C4, and VDRL.

The study protocol was approved by Sultan Qaboos University Ethics Committee with MREC #1903. The study was performed according to the ethical standards laid down in the 1964 Declaration of Helsinki and its later amendments.

### Statistical analyses

Descriptive statistics were used to describe the data. For categorical variables, frequencies and percentages were reported. Differences between groups were analyzed using Pearson’s χ2 tests (or Fisher’s exact tests for cells < 5). For continuous variables, mean and standard deviation were used to summarize the data. Statistical analyses were conducted using STATA version 16.1 (STATA Corporation, College Station, TX, USA).

## Results

The study population included 113 cSLE and 53 controls (45 JIA, 2 MCTD and 6 systemic vasculitis) who fulfilled the inclusion criteria. Table 1 shows the demographic features of the groups demonstrating cSLE cases to have older age at disease onset (*p* < 0.001) with a higher frequency of familial SLE (*p* < 0.001). Table 2 demonstrates the clinical and laboratory criteria for cSLE during the clinical course. Most of these features were significantly higher in cSLE than control group throughout the clinical course. The most common clinical manifestation of cSLE at disease onset in our cohort included arthritis (62%), fever (52%), leukopenia (40%) and proteinuria (39%) while the most common immunological parameters included ANA (89%), dsDNA (64%), and low complements (C3 and C4) (44%) at first visit, first year and last assessment respectively.

**Table 1:** Demographic and clinical and characteristics of cSLE cohort compared to controls

Characteristic, *mean ± SD unless specified otherwise*	All (*N* = 164)	Control (*n* = 51)	Case (*n* = 113)	*p*-value
Age, years	12.8 ± 5.5	10.9 ± 5.2	13.6 ± 5.5	0.004
Female gender, n (%)	123 (75%)	43 (84%)	80 (71%)	0.064
Age at diagnosis, years	6.6 ± 3.5	5.0 ± 3.4	7.3 ± 3.4	< 0.001
Disease duration, years	6.1 ± 4.4	5.7 ± 3.9	6.1 ± 4.6	0.406
Familial, n (%)	47 (29%)	4 (7.8%)	43 (38%)	< 0.001

*SD* Standard deviation

**Table 2:** Clinical and immunological characteristics of the cSLE cohort and the controls at first visit, first year and latest periods

Characteristic, n (%)	First visit			First year visit			Latest visit		
	Controls (*n* = 51)	Cases (*n* = 113)	*p*-value	Controls (*n* = 51)	Cases (*n* = 113)	*p*-value	Controls (*n* = 51)	Cases (*n* = 113)	*p*-value
Fever	6 (12%)	59 (52%)	< 0.001	6 (12%)	60 (53%)	< 0.001	6 (12%)	60 (53%)	< 0.001
Malar rash	0	28 (25%)	< 0.001	0	33 (29%)	< 0.001	0	34 (30%)	< 0.001
Discoid rash	0	5 (4.4%)	0.326	0	7 (6.2%)	0.100	0	7 (6.2%)	0.100
Photosensitivity	0	12 (11%)	< 0.001	0	12 (11%)	0.019	0	12 (11%)	0.019
Oral ulcer	0	17 (15%)	< 0.001	0	18 (16%)	0.001	0	20 (18%)	< 0.001
Alopecia	1 (2.0%)	19 (17%)	0.008	1 (2.0%)	19 (17%)	0.008	1 (2.0%)	20 (18%)	0.004
Arthritis	48 (94%)	70 (62%)	< 0.001	48 (94%)	72 (64%)	< 0.001	48 (94%)	74 (65%)	< 0.001
*Renal disorders*									
Proteinuria, > 0.5 g/day	0	44 (39%)	< 0.001	0	51 (45%)	< 0.001	0	56 (50%)	< 0.001
Cellular cast	1 (2.0%)	11 (9.7%)	0.107	1 (2.0%)	12 (11%)	0.066	1 (2.0%)	15 (13%)	0.023
Lupus nephritis, II-V	0	33 (29%)	< 0.001	0	39 (35%)	< 0.001	0	46 (41%)	< 0.001
Neurological disorders	0	9 (8.0%)	0.058	0	11 (9.7%)	0.018	0	15 (13%)	0.006
Serositis	0	12 (11%)	0.019	0	17 (15%)	0.002	0	21 (19%)	< 0.001
*Hematological disorders*									
Hemolytic anemia	0	30 (27%)	< 0.001	0	30 (27%)	< 0.001	0	33 (29%)	< 0.001
Leukopenia	0	45 (40%)	< 0.001	2 (3.9%)	50 (44%)	< 0.001	2 (3.9%)	52 (46%)	< 0.001
Thrombocytopenia	0	18 (16%)	0.001	0	18 (16%)	0.001	0	18 (16%)	0.001
*Immunological disorders*									
Antinuclear antibody	51 (100%)	100 (89%)	0.010	51 (100%)	104 (92%)	0.058	51 (100%)	105 (93%)	0.059
Anti-dsDNA	3 (5.9%)	72 (64%)	< 0.001	4 (7.8%)	78 (69%)	< 0.001	4 (7.8%)	81 (72%)	< 0.001
Anti-smith	1 (2.0%)	13 (12%)	0.066	1 (2.0%)	15 (13%)	0.023	1 (2.0%)	16 (14%)	0.024
Antiphospholipid	1 (2.0%)	23 (20%)	0.001	1 (2.0%)	26 (23%)	< 0.001	1 (2.0%)	30 (27%)	< 0.001
Direct Coombs test	1 (2.0%)	47 (41%)	< 0.001	1 (2.0%)	48 (42%)	< 0.001	1 (2.0%)	50 (44%)	< 0.001
Low C3 or low C4^a^	2 (3.9%)	31 (27%)	< 0.001	3 (5.9%)	28 (25%)	0.004	3 (5.9%)	28 (25%)	0.004
Low C3 and low C4^a^	0	66 (44%)	< 0.001	0	74 (65%)	< 0.001	0	74 (65%)	< 0.001
Meet ACR 1997	2 (3.9%)	56 (50%)	< 0.001	2 (3.9%)	64 (57%)	< 0.001	2 (3.9%)	74 (65%)	< 0.001
Meet SLICC 2012	3 (5.9%)	86 (76%)	< 0.001	3 (5.9%)	95 (84%)	< 0.001	3 (5.9%)	97 (86%)	< 0.001
Meet ACR/EULAR 2017	4 (7.8%)	92 (81%)	< 0.001	5 (9.8%)	99 (88%)	< 0.001	5 (9.8%)	101 (89%)	< 0.001

Neurological disorders include seizures, psychosis, delirium, neuropathy, or myelitis; VDRL, venereal disease research laboratory; ACR, American College of Rheumatology 1997; SLICC, Systemic Lupus Erythematosus International Collaborating Clinics; EULAR, European League Against Rheumatism

Luekopenia (< 4 × 10^9^/L), thrombocytopenia (< 100 × 10^9^/L), antiphospholipid (ACL (anticardiolipin antibody), B2GP-1 (anti–β2-glycoprotein 1), lupus anticoagulant)

The median disease duration was 5 (2.5–9) years with a range of 1–19 years

^a^These groups are mutually exclusive

**Fig. 1:**
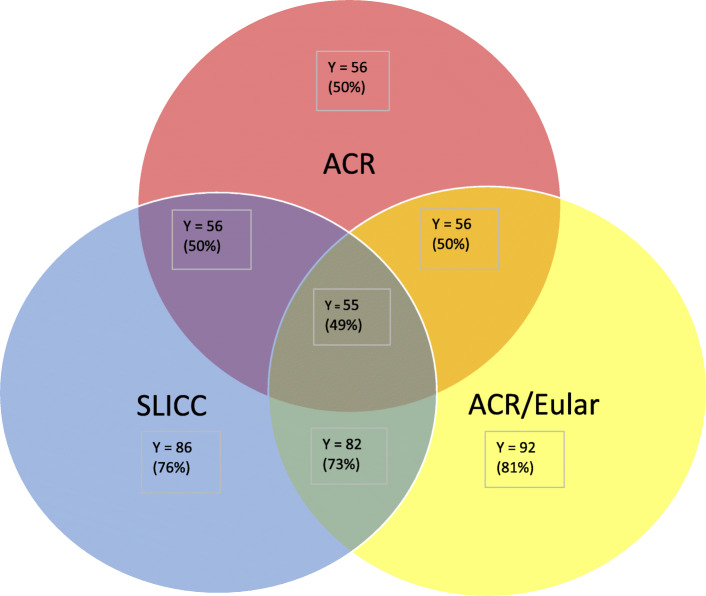
cSLE (*n* = 113) patients classified according to ACR 1997, SLICC 2012, EULAR/ACR 2019 classification criteria

Figure 1 demonstrates the number of cSLE patients that fulfilled the classifications criteria at first visit. A total of 49% of cSLE cohort fulfilled the 3 classification criteria, while all the patients who fulfilled the ACR 1997 also fulfilled the SLICC criteria and ACR/EULAR 2019 criteria. Significant overlap was also demonstrated between SLICC 2012 and ACR/EULAR 2019 criteria with the two criteria correctly classifying 88% of cSLE patients. Of the patients who fulfilled the SLICC 2012 criteria but not the ACR 1997 criteria (30/57), the common manifestations in this cohort were fever (63%; *n* = 19), arthritis (40%; *n* = 12), nephritis (23%; *n* = 7) and proteinuria (20%; *n* = 6) while the most common immunological parameters included dsDNA (63%; *n* = 19), positive VDRL (47%; *n* = 14) and low complements (C3 and C4) (47%; *n* = 14) at first visit. Of the patients who fulfilled the EULAR/ ACR 2019 criteria but not the ACR 1997 criteria (37/57), the common manifestation in this cohort were fever (62%; *n* = 23), arthritis (49%; *n* = 18), nephritis (16%; *n* = 6) and proteinuria (14%; *n* = 5) while the most common immunological parameters included dsDNA (62%; *n* = 23), positive VDRL (49%; *n* = 18) and low complements (C3 and C4) (51%; *n* = 19) at first visit. In total, there were 13 patients diagnosed with cSLE despite having negative ANA in our study. Of the patients diagnosed with ANA negative cSLE, 6 patients presented with low complements and lupus nephritis (class III/IV), while 7 patients presented with hypocomplementemic urticarial vasculitis as initial manifestation of cSLE.

The ACR 1997 criteria was met in 50, 57, and 65% of cSLE patients, while the median number of ACR criteria there were met were 3 (2–5), 4 (3–5), and 4 (3–5) criteria at first visit, first year and last assessment, respectively. The SLICC criteria was met in 76, 84, 86% of cSLE patients, while the median number of SLICC criteria there were met were 5.5 (3–7), 6 (4–8), 6 (4–8) at first visit, first year and last assessment respectively. While, the EULAR/ACR criteria was met in 81, 88 and 89% of cSLE patients, while median weight scores were 19 (13–26), 21 (15–28) and 23 (16–28) at first visit, first year and last assessment respectively as shown in Table 2.

Table 3 summarizes the performance characteristics of the 1997 ACR, SLICC, and the EULAR/ACR criteria in terms of sensitivity, specificity, predictive values and accuracy. The SLICC 2012 criteria has a greater sensitivity compared to ACR 1997 at first visit (*p* < 0.001), first year (*p* < 0.001) and last follow-up (*p* < 0.001), respectively. However, there was no significant differences in specificity between the ACR 1997 and SLICC 2012 criteria at neither first visit (*p* = 1.000), at first year (*p* = 1.000) nor at last follow-up (*p* = 1.000). The ACR/EULAR 2019 criteria (at a cut off score of ≥13 as compared to the traditional cut of score of ≥10) has a greater sensitivity compared to ACR 1997 at first visit (*p* < 0.001), first year (*p* < 0.001) and last follow-up (*p* < 0.001), respectively. No significant differences were observed with regards to specificity between the ACR 1997 and ACR/EULAR 2019 criteria at neither first visit (*p* = 1.000), at first year (*p* = 1.000) nor at last follow-up (*p* = 1.000). There were, however, no significant differences in sensitivity between the ACR/EULAR 2019 (at a cut off score of ≥13 as compared to the traditional cut of score of ≥10) and SLICC 2012 criteria at first visit (*p* = 1.000), first year (*p* = 1.000) and last follow-up (*p* = 1.000), respectively. No significant differences were observed with regards to specificity between the ACR/EULAR 2019 and SLICC 2012 criteria at neither first visit (*p* = 1.000), at first year (*p* = 1.000) nor at last follow-up (*p* = 1.000).

**Table 3:** Performance measures for the ACR 1997, SLICC 2012 and ACR/EULAR 2019 criteria according to first visit, first year and latest periods

Criteria	Sensitivity (95% CI)	Specificity (95% CI)	PPV (95% CI)	NPV (95% CI)	ROC (95% CI)	Accuracy (95% CI)
ACR 1997						
1st visit	49% (40–59%)	96% (87–99%)	97% (88–99%)	46% (36–56%)	0.73 (0.67–0.78)	64% (56–71%)
1st year	57% (47–66%)	96% (87–99%)	97% (90–99%)	50% (40–60%)	0.76 (0.71–0.82)	69% (61–76%)
Latest	66% (56–74%)	96% (87–99%)	97% (91–99%)	56% (45–66%)	0.81 (0.76–0.86)	75% (68–81%)
SLICC						
1st visit	76% (67–84%)	94% (84–99%)	97% (91–99%)	64% (52–75%)	0.85 (0.80–0.90)	82% (75–87%)
1st year	84% (76–90%)	94% (84–99%)	97% (91–99%)	73% (60–83%)	0.89 (0.84–0.94)	87% (81–92%)
Latest	86% (78–92%)	94% (84–99%)	97% (92–99%)	75% (63–85%)	0.90 (0.85–0.95)	88% (83–93%)
ACR/EULAR						
1st visit	81% (73–88%)	92% (81–98%)	96% (90–99%)	69% (57–80%)	0.87 (0.82–0.92)	85% (78–90%)
1st year	88% (80–93%)	90% (79–97%)	95% (89–98%)	77% (64–87%)	0.89 (0.84–0.94)	88% (83–93%)
Latest	89% (82–94%)	90% (79–97%)	95% (89–99%)	79% (67–89%)	0.90 (0.85–0.95)	90% (84–94%)
ACR/EULAR^a^						
1st visit	76% (67–84%)	96% (87–99%)	98% (92–99%)	65% (53–75%)	0.86 (0.81–0.91)	83% (76–88%)
1st year	83% (74–89%)	96% (87–99%)	98% (93–99%)	71% (59–81%)	0.89 (0.85–0.94)	87% (81–92%)
Latest	84% (76–90%)	96% (87–99%)	98% (93–99%)	73% (61–83%)	0.90 (0.86–0.94)	88% (83–93%)

ACR/EULAR^a^, a score of ≥13 as compared to the traditional cut of score of ≥10

The median disease duration was 5 (2.5–9) years with a range of 1–19 years

*ACR* American College of Rheumatology 1997, *SLICC* Systemic Lupus Erythematosus International Collaborating Clinics, *EULAR* European League Against Rheumatism, *CI* Confidence interval, *PPV* Positive predictive value, *NPV* Negative predictive value, *ROC* Area under the received operating curve

## Discussion

The ACR 1997, SLICC 2012 and EULAR/ACR 2019 classification criteria were validated based on adult patients. To date, there are no classification criteria specific for children with SLE. Thus, it was necessary to assess the performance of the existing classification criteria in cSLE. Similarly, it is essential to assess the performance among various population knowing that the clinical manifestations of SLE show substantial geographical and/or ethnic variation due to genetic and non-genetic factors that influence disease expression [20]. To the best of our knowledge, this is the first study to have assessed the performance among the three classification criteria originating from children of Arab ethnicity from the Middle East.

In our cSLE cohort, the most common clinical manifestations at disease onset were arthritis (62%), fever (52%), proteinuria > 0.5 g.day (39%) and leukopenia (40%). The result of our cohort correlates with a recent meta-analysis that highlights that these major organ involvement are more common in cSLE compared to adult onset SLE [21]. However, compared to other cSLE cohorts described in the literature, our cSLE cohort had a much younger age of disease onset of 7 (4–10) years compared to median age 10–13 years in other studies [13–19]. Perhaps, the lower age of transition to adult care in Arab health care due to cultural differences plays a role in having a younger cohort. Similarly, a higher percentage of familial SLE (38%) was noted in our cohort than that reported from studies of multiplex families from Western countries, with 5–12% having a relative with SLE [22]. This is most likely due to higher degree of consanguineous marriage in Oman reaching 36% in addition to 20% of marriages being contracted between specific tribal groups [23]. However, despite this relatively high rate of familial SLE in our cohort, the clinical and serological manifestations of familial and non-familial SLE are similar, representing a comparable disease entity in our population [24].

With regards to the performance of the 3 classification criteria in our cohort, the EULAR/ACR-2019 criteria had the highest sensitivity (81, 88, 89%) compared to ACR 1997 (49, 57, 66%) and SLICC 2012 (76, 84,86%) at first visit, first year and last assessment, respectively; while the ACR 1997 had the highest specificity (96%) compared to SLICC 2012 (94%) and EULAR/ACR 2019 (92, 90, 90%) at first visit, first year and last assessment, respectively. When we increased the threshold score to ≥13 rather than the traditional score ≥ 10 for ACR/EULAR 2019, there was increased specificity (96%) at the expense of lower sensitivity (76, 83, and 84%) at first visit, first year and last assessment.

To date, there has been two other study populations in children comparing the performance of the three classification criteria’s (ACR-1997, SLICC-2012 and EULAR/ACR-2019) [16, 18]. Batu et al described the performance of the 3 classification criteria in a large cohort of cSLE (*n* = 262) from Turkey at diagnosis, reporting sensitivity of SLICC-2012 to be highest (95.4%) compared to ACR-1997 (68.7%) and EULAR/ACR-2019 (91.5%) while the specificity for ACR-1997 (94.8%) to be the highest compared to for SLICC-2012 (89.7%) and EULAR/ACR-2019 (88.5%) [16]. Fonesca et al described the performance of the 3 classifications in cSLE cohort (*n* = 122) from Brazil, at diagnosis and at 1 year follow up. Similarly, they reported the highest sensitivity with SLICC 2012 at diagnosis and 1 year follow up (89.3%, 97,5%) compared to ACR 1997 (70.5, 95.1%) and EULAR/ACR 2019 (87.7%, 95.1); while the specificity of ACR 1997 was highest at diagnosis and 1 year follow up (83.2, 76.4%) compared to SLICC 2012 (80.9, 76.4%) and EULAR/ACR 2019 (67.4, 58.4%). In contrast, to the two previous studies from Turkey and Brazil, our study from Middle East in children with Arab ethnicity, showed the highest sensitivity with the EULAR/ACR 2019 at diagnosis, 1 year follow up and last assessment compared to ACR 1997 and SLICC 2012. Similar to the two studies, the highest specificity was observed with ACR 1997, however the degree of specificity was much higher (96%) compared to the Brazilian cohort (83.2, 76.4%) at first visit and first year follow up, but comparable to Turkish cohort (94.8%) at first visit. Comparable to the results of our cohort was unpublished data from South Indian cSLE cohort (*n* = 107), showing highest performance of sensitivity with the ACR/EULAR 2019 (96.3%) compared to ACR 1997 (73.8%) and SLICC 2012 (94.4%) criteria. The data also demonstrated the highest specificity with ACR 1997 (100%) compared to ACR/EULAR 2019 (98.1%) and SLICC 2012 (96.2%) [24]. Perhaps ethnic, race and geographical differences influence SLE disease expression thereby making certain classification more sensitive than others [25].

Previous cSLE studies comparing the two classification criteria (ACR 1997 to SLICC 2012) demonstrated that SLICC 2012 classifies patients earlier than ACR 1997 criteria. Fonseca et al. assessed the performance of the classification of cSLE at the first visit and at 1 year follow up in a Brazilian center. The sensitivity of SLICC 2012 was higher than for ACR 1997, 82.7% vs 58.0% (*p* < 0.001) at first visit and 96.3% vs 91.3% (*p* = 0.125) at first year, however, specificity was not significantly different [14]. In the multicenter European study, Sag et al., evaluated the performance at time of diagnosis, the sensitivity of SLICC 2012 was higher (98.7% vs 85.3%; *p* < 0.001) but specificity was lower (76.6% vs 93.4% *p* < 0.001) compared to ACR 1997 [13]. Within the UK JSLE cohort, SLICC 2012 was also more sensitive, both at diagnosis (92.9% vs 84.1%) and follow-up (100% vs 92%) [15]. In a study conducted in cSLE (*n* = 86) cohort in Oman, at disease onset 80% (68/86) fulfilled the ACR criteria, while 99% (85/86) fulfilled the SLICC criteria [3].

More recent cSLE studies comparing the two classification criteria (SLICC-2012 and EULAR/ACR- 2019) demonstrated that the sensitivity of SLICC-2012 criteria was higher when compared to ACR/EULAR-2019 at first and last visit (98% vs 94%, first visit, and 98% vs 96%, last visit; *p* < 0.001); while the specificity of ACR/EULAR-2019 was higher when compared to SLICC-2012 at first and last visit (77% vs 67%, first visit, and 81% vs 71%, last visit; *p* < 0.001) in the UK JSLE Cohort Study. Interestingly, another recent study from Israel found the sensitivity and specificity of ACR/EULAR-2019 to be comparable to that of SLICC-2012 at 12 months, between 1 and 2 years, and > 2 years from disease onset [19].

This study is not without limitations. The study is limited by its retrospective design. Also, the cSLE cohort in Oman has distinctive epidemiological features that have been described previously. The cSLE cohort included, had disease onset prior to 13 years of age, making them relatively younger than previous studies. Furthermore, the cohort included had a higher degree of familial SLE than previous studies due to higher degree of consanguineous marriage in the region. We have been able to identify a genetic mutation in a subgroup of these patients with familial SLE in the region (DNASE1L3) [23, 26, 27]. Both these epidemiological factors should be taken into consideration prior to making a generalization on a wider cSLE cohort from other ethnicity. The other major limitations was the relatively low number of controls with diverse differential diagnosis. We were limited with inclusion of controls who were ANA positive. As a result, most of our controls had the diagnosis of JIA. We had only two patients that were misclassified as SLE at initial diagnosis, one with MTCD and other with systemic vasculitis, hence resulting in the much higher specificity results compared to previous studies. However, one of the advantages of the study, was that the data was data was extrapolated by trained physicians with experience in pediatric rheumatology, hence minimizing methodological limitations. Furthermore, the data was compared, over the duration of clinical course, hence giving us the ability to compare the sensitivity and specificity over longer duration than previous studies.

A recent meta-analysis concluded that in cSLE, ACR 1997 had the best overall classification performance based on higher specificity in comparison with SLICC 2012, despite the finding that SLICC 2012 has much higher sensitivity and classifies patients earlier in their disease course than ACR1997 [28]. However, in our cohort of patients from Oman, we can conclude that the ACR/EULAR 2019 criteria, especially with a cut off score of ≥13 rather than the traditional score ≥ 10, had the best overall classification performance based on a higher sensitivity and a comparable specificity to ACR 1997. Given the children have a more fulminant disease onset, clinical course and cumulative damage than adults with SLE, further multinational studies are needed to validate the appropriate cut off score for the newly proposed ACR/EULAR 2019 classification criteria in cSLE to increase early sensitivity and specificity for cSLE classification.

## Conclusion

Our study highlights that there are various trends in performance of various SLE classification criteria among various studies [13–19]. Ethnicity and genetic ancestry play a major role in clinical presentation and outcome of SLE [20]. Hence, it would be pivotal to evaluate the various classification criteria in various ethnicities given the diversity of SLE.

## Data Availability

The datasets used and/or analyzed during the current study are available from the corresponding author on.

## Abbreviations

ACR: American College of Rheumatology
SLICC: Systemic Lupus International Collaborating Clinics
EULAR/ACR: European League Against Rheumatism (EULAR) and ACR
SLE: systemic lupus erythematosus
cSLE: childhood onset SLE

## References

[1] Tarr T, Dérfalvi B, Győri N, Szántó A, Siminszky Z, Malik A, Szabó AJ, Szegedi G, Zeher M (2015). Similarities and differences between pediatric and adult patients with systemic lupus erythematosus. Lupus..

[2] Fonseca R, Aguiar F, Rodrigues M, Brito I (2018). Clinical phenotype and outcome in lupus according to age: a comparison between juvenile and adult onset. Reumatol Clin.

[3] Al Rasbi A, Abdalla E, Sultan R, Abdullah N, Al Kaabi J, Al-Zakwani I (2018). Spectrum of systemic lupus erythematosus in Oman: from childhood to adulthood. Rheumatol Int.

[4] Bertsias GK, Pamfil C, Fanouriakis A, Boumpas DT (2013). Diagnostic criteria for systemic lupus erythematosus: has the time come?. Nat Rev Rheumatol.

[5] Hochberg MC (1997). Updating the American College of Rheumatology revised criteria for the classification of systemic lupus erythematosus. Arthritis Rheum.

[6] Smith EL, Shmerling RH (1999). The American College of Rheumatology criteria for the classification of systemic lupus erythematosus: strengths, weaknesses, and opportunities for improvement. Lupus..

[7] Petri M, Magder L (2004). Classification criteria for systemic lupus erythematosus: a review. Lupus..

[8] Petri M (2005). Review of classification criteria for systemic lupus erythematosus. Rheum Dis Clin N Am.

[9] Tedeschi SK, Johnson SR, Boumpas D, Daikh D, Dörner T, Jayne D, Kamen D, Lerstrøm K, Mosca M, Ramsey-Goldman R, Sinnette C, Wofsy D, Smolen JS, Naden RP, Aringer M, Costenbader KH (2018). Developing and refining new candidate criteria for systemic lupus erythematosus classification: an international collaboration. Arthritis Care Res (Hoboken).

[10] Petri M, Orbai AM, Alarcón GS, Gordon C, Merrill JT, Fortin PR, Bruce IN, Isenberg D, Wallace DJ, Nived O, Sturfelt G, Ramsey-Goldman R, Bae SC, Hanly JG, Sánchez-Guerrero J, Clarke A, Aranow C, Manzi S, Urowitz M, Gladman D, Kalunian K, Costner M, Werth VP, Zoma A, Bernatsky S, Ruiz-Irastorza G, Khamashta MA, Jacobsen S, Buyon JP, Maddison P, Dooley MA, van Vollenhoven RF, Ginzler E, Stoll T, Peschken C, Jorizzo JL, Callen JP, Lim SS, Fessler BJ, Inanc M, Kamen DL, Rahman A, Steinsson K, Franks AG, Sigler L, Hameed S, Fang H, Pham N, Brey R, Weisman MH, McGwin G, Magder LS (2012). Derivation and validation of the systemic lupus international collaborating clinics classification criteria for systemic lupus erythematosus. Arthritis Rheum.

[11] Tedeschi SK, Johnson SR, Boumpas DT, Daikh D, Dörner T, Diamond B, Jacobsen S, Jayne D, Kamen DL, McCune WJ, Mosca M, Ramsey-Goldman R, Ruiz-Irastorza G, Schneider M, Urowitz M, Wofsy D, Smolen JS, Naden RP, Aringer M, Costenbader KH (2019). Multicriteria decision analysis process to develop new classification criteria for systemic lupus erythematosus. Ann Rheum Dis.

[12] Aringer M, Costenbader K, Daikh D, Brinks R, Mosca M, Ramsey-Goldman R, Smolen JS, Wofsy D, Boumpas DT, Kamen DL, Jayne D, Cervera R, Costedoat-Chalumeau N, Diamond B, Gladman DD, Hahn B, Hiepe F, Jacobsen S, Khanna D, Lerstrøm K, Massarotti E, McCune J, Ruiz-Irastorza G, Sanchez-Guerrero J, Schneider M, Urowitz M, Bertsias G, Hoyer BF, Leuchten N, Tani C, Tedeschi SK, Touma Z, Schmajuk G, Anic B, Assan F, Chan TM, Clarke AE, Crow MK, Czirják L, Doria A, Graninger W, Halda-Kiss B, Hasni S, Izmirly PM, Jung M, Kumánovics G, Mariette X, Padjen I, Pego-Reigosa JM, Romero-Diaz J, Rúa-Figueroa Fernández Í, Seror R, Stummvoll GH, Tanaka Y, Tektonidou MG, Vasconcelos C, Vital EM, Wallace DJ, Yavuz S, Meroni PL, Fritzler MJ, Naden R, Dörner T, Johnson SR (2019). 2019 European League Against Rheumatism/American College of Rheumatology classification criteria for systemic lupus erythematosus. Ann Rheum Dis.

[13] Sag E, Tartaglione A, Batu ED, Ravelli A, Khalil SM, Marks SD (2014). Performance of the new SLICC classification criteria in childhood systemic lupus erythematosus: a multicentre study. Clin Exp Rheumatol.

[14] Fonseca AR, Gaspar-Elsas MI, Land MG, de Oliveira SK (2015). Comparison between three systems of classification criteria in juvenile systemic lupus erythematous. Rheumatology (Oxford).

[15] Lythgoe H, Morgan T, Heaf E, Lloyd O, Al-Abadi E, Armon K (2017). UK JSLE study group. Evaluation of the ACR and SLICC classification criteria in juvenile-onset systemic lupus erythematosus: a longitudinal analysis. Lupus..

[16] Rodrigues Fonseca A, Felix Rodrigues MC, Sztajnbok FR, Gerardin Poirot Land M, Knupp Feitosa de Oliveira S (2019). Comparison among ACR1997, SLICC and the new EULAR/ACR classification criteria in childhood-onset systemic lupus erythematosus. Adv Rheumatol.

[17] Tao JJ, Hiraki LT, Levy DM, Silverman ED (2019). Comparison of sensitivities of American College of Rheumatology and Systemic Lupus International Collaborating Clinics Classification Criteria in childhood-onset systemic lupus erythematosus. J Rheumatol.

[18] Batu ED, Akca UK, Kısaarslan AP, Sağ E, Demir F, Demir S (2021). The Performances of the ACR 1997, SLICC 2012, and EULAR/ACR 2019 Classification Criteria in Pediatric Systemic Lupus Erythematosus. J Rheumatol..

[19] Levinsky Y, Broide M, Kagan S, Goldberg O, Scheuerman O, et al. Performance of 2019 EULAR/ACR classification criteria for Systemic Lupus Erythematosus in a pediatric population - a multicenter study. Rheumatology (Oxford). 2021:keab140. 10.1093/rheumatology/keab140. 10.1093/rheumatology/keab140 33560345

[20] González LA, Toloza SM, McGwin G, Alarcón GS (2013). Ethnicity in systemic lupus erythematosus (SLE): its influence on susceptibility and outcomes. Lupus..

[21] Bundhund PK, Kumari A, Huang F (2017). Differences in clinical features observed between childhood-onset versus adult-onset systemic lupus erythematosus: a systematic review and meta-analysis. Medicine (Baltimore).

[22] Arnett FC, Shulman LE (1976). Studies in familial systemic lupus erythematosus. Medicine (Baltimore).

[23] Abdwani R, Hira M, Al-Nabhani D, Al-Zakwani I (2011). Juvenile systemic lupus erythematosus in the Sultanate of Oman: clinical and immunological comparison between familial and non-familial cases. Lupus..

[24] Nair HB, Balan S (2020). Validation of 2019 EULAR/ACR classification criteria for SLE in South Indian Juvenile SLE cohort. P140. Rheumatology.

[25] Lewis MJ, Jawad AS (2017). The effect of ethnicity and genetic ancestry on the epidemiology, clinical features and outcome of systemic lupus erythematosus. Rheumatology (Oxford).

[26] Al-Mayouf S, Sunker A, Abdwani R, Abrawi SA, Almurshedi F, Alhashmi N (2011). Loss-of-function variant in *DNASE1L3* causes a familial form of systemic lupus erythematosus. Nat Genet.

[27] Al-Mayouf SM, Abdwani R, Al-Brawi S (2012). Familial juvenile systemic lupus erythematosus in Arab children. Rheumatol Int.

[28] Hartman EAR, van Royen-Kerkhof A, Jacobs JWG, Welsing PMJ, Fritsch-Stork RDE (2018). Performance of the 2012 systemic lupus international collaborating clinics classification criteria versus the 1997 American College of Rheumatology classification criteria in adult and juvenile systemic lupus erythematosus. A systematic review and meta-analysis. Autoimmun Rev.

