# Correction to: Evaluating the performance of ACR, SLICC and EULAR/ACR classification criteria in childhood onset systemic lupus erythematosus

**DOI:** 10.1186/s12969-021-00655-6

**Published:** 2021-12-02

**Authors:** Reem Abdwani, Eman Al Masroori, Eiman Abdullah, Safiya Al Abrawi, Ibrahim Al-Zakwani

**Affiliations:** 1 Department of Child Health, College of Medicine & Health Sciences, Sultan Qaboos University, Muscat, Oman; 2 Department of Pediatrics, Royal Hospital, Muscat, Oman; 3 Department of Pharmacology & Clinical Pharmacy, College of Medicine & Health Sciences, Sultan Qaboos University, Muscat, Oman; 4 Gulf Health Research, Muscat, Oman

**Correction to: Pediatr Rheumatol 19, 141 (2021)**

**https://doi.org/10.1186/s12969-021-00619-w**

Following publication of the original article [1], the author identified an error in the author name of Eman Al Masroori.

- The incorrect author name is: Eiman Masroori
- The correct author name is: Eman Al Masroori

The author group has been updated above and the original article [1] has been corrected.
